# Supplementary material for: Care navigation addresses issues of tele-mental health acceptability and uptake in rural and remote Australian communities
Source: PLoS One. 2024 Apr 4;19(4):e0298655. doi: 10.1371/journal.pone.0298655 (PMC10994303; doi:10.1371/journal.pone.0298655)
Supplement: S1 File — (DOCX) [file pone.0298655.s001.docx]

Isaac Navicare Evaluation Semi-Structured Interview Guide

**Before the interview**

|  | Check if the participant has had a chance to review the participant information form, and ask if they have any questions prior to the interview |
| --- | --- |
|  | Explain that no questions will be asked about their mental health status, or the mental health status of anyone they may have discussed with the Care Navigator, such as a child or family member |
|  | Explain that the interview will be audio recorded and check if the participant agrees to being recorded |
|  | Confirm the expected interview duration with the participant and check whether they have a time they need to finish by, e.g., for another appointment |
|  | Let the participant know that they will have an opportunity at the end of the interview to add anything they would like to, and they are welcome to add any additional thoughts within two weeks of the interview by contacting the interviewer – additional information can be provided by email, phone or video conference as per the participant’s preference. |
|  | ***Commence audio recording*** |
|  | ***Obtain verbal consent to participate in the interview*** |

**Interview Questions**

*Interviews will be conducted in a semi-structured format. The wording of individual questions may change, and probing questions will be asked as needed to gain further information about key points. The overall topics and aims will remain the same.*

1. [Reach] How did you find out about Isaac Navicare?
2. [Reach] When you first had contact with Isaac Navicare were you seeking support for yourself or for a family member, friend, colleague or someone you care for?
3. [Effectiveness] Without describing your/other person’s mental health status, can you explain the type of support you received from the Care Navigator? For example, referral to a local treatment service, referral to an online treatment service, referral to local community supports such as emergency financial aid, housing, a social worker, allied health or legal support?
4. [Effectiveness] Did you receive follow-up contact from the Care Navigator?
5. [Effectiveness] (if yes) Please describe the type of follow up you received e.g., were there issues booking an appointment?
6. [Effectiveness] (if yes) Was that follow-up contact helpful?
7. [Effectiveness] Did you/ the person you contacted Navicare about act on the referral to the service/s recommended? For example, attending an appointment with a service provider
   1. [Effectiveness] Why/why not?
8. [Effectiveness] If Navicare didn’t exist at the time you contacted the Care Navigator, what would you have done instead?
   1. (Potential follow up) Has/ how has Navicare improved access to mental health services in your opinion?
   2. (Potential follow up) How long had you been seeking support for your/ other person’s mental health prior to being in contact with the Care Navigator?
9. [Implementation] Are you aware of the supported telehealth site at Moranbah?
10. [Implementation] Is supported telehealth (going to the Navicare offices to attend a virtual appointment) something that you did/ would find helpful? Why/ why not?
    1. (Potential follow-up) Has your level of comfort using telehealth/ video conferencing changed during the COVID-19 pandemic?
11. [Reach/Implementation] We’re interested to hear about people who are missing out on being able to access mental health supports. Are there any groups of people you can think of in your area who might need mental health supports and are missing out? (For example, people who don’t have reliable internet access, people who speak English as a second language.)
    1. (Potential follow up) Do you have any thoughts on how to better engage with or support these hard-to-reach groups?
12. [Effectiveness/Implementation] What is the best or most helpful thing about the Isaac Navicare service?
13. [Implementation/Maintenance] What improvements would you suggest?
14. [Effectiveness/Implementation/Maintenance] Would you use Isaac Navicare again if you needed to?
15. Is there anything else you would like to add?

**At the end of the interview**

|  | Thank the participant for their time and feedback |
| --- | --- |
|  | Remind the participant that they are welcome to clarify their statements or add any additional information by contacting the interviewer by email within two weeks following the interview – their comments will be added to the transcript |
|  | ***Upload the audio recording to the server and delete from recording device*** |
