# Supplementary material for: Care navigation addresses issues of tele-mental health acceptability and uptake in rural and remote Australian communities
Source: PLoS One. 2024 Apr 4;19(4):e0298655. doi: 10.1371/journal.pone.0298655 (PMC10994303; doi:10.1371/journal.pone.0298655)
Supplement: Attachment — Submitted filename: Response_to_Reviewers_Nov_2023.docx [file pone.0298655.s002.docx]

Response to Reviewers

Nov 2023

| Comment | Response | Line/s |
| --- | --- | --- |
| Editor | | |
| More clarity needed around the financial statement and any potential competing interest. | Thank you for identifying that we had not been clear about the relationship between the funder and the research team. We have updated the funding statement to clarify that the funder had no direct role in the research other than funding two authors’ salaries. We added an author contribution statement and a competing interests statement declaring the commercial affiliation and clarifying that this relationship does not alter our adherence to all PLOS ONE policies on sharing data and materials. | 13-22  34-39 |
| Formatting of manuscript and file name | The formatting has been updated to meet PLOS ONE’s style requirements, including those for file naming. | Throughout |
| Reviewer 1 | | |
| The study title needs to include the geographical location (country) of the study. | The title has been amended. | 1-2 |
| (Abstract) Navicare need to be defined or explained in the introduction. | Text has been added to the abstract. | 57 |
| (Abstract) The authors need to define the evidence gap and the study aim in the introduction. | Text has been added to the abstract. | 59-61 |
| The authors need define the study participants. | Text has been added to the methods section. | 246-252  260 |
| The authors need to also indicate the end date. | Text has been added to the methods section to clarify that database records were collected for all clients between 9 November 2021 and 8 November 2022, and survey and interview data collection was conducted between December 2022 and March 2023. | 230-231  251-252 |
| The authors should consider deleting discussion as a heading and integrating the information to the conclusion as policy recommendation/s. | We appreciate this suggestion and have added policy recommendations to the conclusion which we agree have improved this paper.  We have considered Reviewer 1’s proposal to combine the discussion and conclusion sections alongside Reviewer 2’s suggestions for additions and improvements to the discussion section. We have decided to retain and improve the discussion section based on Reviewer 2’s recommendations and enhanced the conclusion with the additional policy recommendations text based on Reviewer 1’s recommendation. | 464-482  495-502  524-529 |
| The phrase after the reference at line 77 also need referencing. | Reference added | 94 |
| The sentence at line 87 need to be referenced. | Reference added | 104 |
| The sentence at line 97 need to be referenced. | Reference added | 114 |
| The sentence at line 101 need to be referenced. | Text amended to clarify that the references prior to this sentence also relate to this sentence. | 118-119 |
| LGBTQIA+ at line 103 need to be written in full on first use and put in brackets (LGBTQIA+) | Text amended as requested | 121-122 |
| The sentence at line 109 ending with via telehealth, need to be referenced. | This sentence is a direct quote taken from the conclusion of a review paper by Mseke et al. (2023, reference 21). The reference has been included at the end of the quote. | 127-130 |
| The sentence at line 112 need to be referenced | Text amended to clarify that the references in the following sentence relate to both sentences. | 131-134 |
| At line 125, authors mentioned studies as evidence but referenced only one study (36). | Text amended to clarify that this reference is a review paper including multiple studies. | 143-145 |
| Reviewer 2 | | |
| The introduction paragraph contains helpful information for context however should be revised slightly for an international audience. i.e. it is not clear that this paragraph is focused on Australia. | Text has been amended. | 91 |
| Line 132 – the Bowen Basin region is first introduced here – it would be helpful for the reader to give an indication of where this is. | Text has been added to clarify this. | 162 |
| Line 139 – Please clarify what is meant by a supported telehealth site – it is mentioned in a few areas of the paper but it is unclear for the reader what this is. | Text has been added to clarify this. | 170-173 |
| Line 140 – what is meant by ‘non-stigmatising’ location? | Text has been added to clarify this. | 175-178 |
| Line 144 – Please clarify if the agricultural workforce are resident or non-resident. | Text has been amended | 180-182 |
| Line 186 – The RE-AIM Framework citation is missing. | Citation added | 224 |
| Line 191 – Was ethic approval needed/obtained for use of the database in the evaluation? | Text has been amended to clarify that the ethical approval covered all data used in the evaluation including the database. | 222-223 |
| Line 201 – What is the Bridging Study? This seems like something separate from this evaluation study – please clarify or remove the statement. | The text relating to The Bridging Study was included to clarify that there is a separate Navicare study with a different research team which is currently underway. The pilot study reported in this manuscript was conducted prior to the Bridging Study, which focuses on expansion of Navicare into other areas of the Bowen Basin region of Queensland.  There is potential for confusion between the pilot study and The Bridging Study. It is important to clarify for participants of both studies, for the local community, and for the two separate funding bodies, therefore it is important to distinguish them. We have amended the text to clarify this. | 240-243 |
| Line 203 – Consider using the heading ‘Recruitment’ or similar | Text amended accordingly. | 277 |
| Line 204 is repeated in Line 213 | Repetitive text removed from line 213. | 245-246 |
| Line 214 – Did anyone take up the option to share a reflection? I don’t think I saw this in the findings and will be useful for the reader to include in the findings section and understand how this was incorporated into the analysis, if relevant. | The following text was added: “No interview participants took up the offer of sharing a reflection in the two weeks following their interview.” | 291-292 |
| Line 244 – a brief overview of interview participant characteristics/demographics would be useful for the reader. | Descriptive text about the interview participants has been included in the results section. | 292-297 |
| The quotes are well chosen and convey the findings well. A descriptor at the end of each quote would further strengthen the use of quotes – e.g. parent | Descriptors have been added throughout the results section. | Results section |
| Line 278 – the 276 referrals relate to referrals made by the care navigator. Please include this information so the reader can easily distinguish this from referring providers to the Navicare service. | The text has been amended accordingly. | 330 |
| Table 2 – I note that this table has a total of 32 ‘did not refer’. But in Table 3, the count is 41. Is there an error? | Table 2 vs Table 3: In Table 2, ‘Did not refer’ relates to all referrals, both for mental health and wrap-around supports. Table 3 relates to referrals to mental health supports only. Text amended immediately prior to Table 2 to clarify ‘Did not refer’ means for any support, either mental health or wrap around support. | 333-334 |
| Line 291 – Is there a maximum number of weeks that could be added here? | This text has been amended for clarification. The maximum wait time described was two months without receiving a response from a service provider that the care navigator had referred the participant to. | 344-346 |
| Line 400 – At least 11% of clients identified as First Nations – this is important and I suggest that the authors consider inclusion of this as a discussion point. | This is an excellent point, and we have added text discussing this, and possible opportunities for future research, in the discussion section. | 464-474 |
| Line 431 – Please consider - ‘reduced’ waiting times or ‘short’ waiting times? | Text amended accordingly | 519 |
| The article describes a rigorous evaluation study of a model of care that has increased access to mental health care. Engagement with the existing literature about similar care navigation models for mental health would further strengthen this article. What does this study add? What are the key learnings? | Thank you for identifying this important omission. We have added text to the introduction and the discussion which addresses previous care navigation literature and the contribution of this study. | 151-158  495-502 |
